# Chemotherapy-associated oral microbiome changes in breast cancer patients

**DOI:** 10.3389/fonc.2022.949071

**Published:** 2022-08-09

**Authors:** Ingeborg Klymiuk, Ceren Bilgilier, Alexander Mahnert, Andreas Prokesch, Christoph Heininger, Ingeborg Brandl, Hanka Sahbegovic, Christian Singer, Thorsten Fuereder, Christoph Steininger

**Affiliations:** ^1^ Division of Cell Biology, Histology and Embryology, Gottfried Schatz Research Center, Medical University of Graz, Graz, Austria; ^2^ Division of Infectious Diseases and Tropical Medicine, Department of Medicine I, Medical University Vienna, Vienna, Austria; ^3^ Diagnostic and Research Institute of Hygiene, Microbiology and Environmental Medicine, Medical University of Graz, Graz, Austria; ^4^ Department of Gynecology, Clinical Department of General Gynecology and Gynecological Oncology. Medical University Vienna, Vienna, Austria; ^5^ Division of Oncology, Department of Medicine I, Medical University Vienna, Vienna, Austria; ^6^ Karl Landsteiner Institute for Microbiome Research, St. Pölten, Austria

**Keywords:** chemotherapy, oral microbiome, oral side effects, *Actinomyces*, microbial pattern, 16S rRNA gene amplicon analysis

## Abstract

Cytotoxic chemotherapy with or without a combination of humanized monoclonal antibodies is regarded as the gold standard of personalized medicine for the treatment of breast cancer patients. Significant medication-related side effects are common accompanying phenomena for these patients, such as oral discomfort, mucositis, or even osteonecrosis of the jaw. In this study, we analyze the saliva samples of 20 breast cancer patients at three time points throughout their chemotherapy: at the baseline prior to treatment initiation (T1), after four-to-six cycles of chemotherapy (T2), and 1 year after the start of the treatment (T3) to investigate and characterize the long-term effects of chemotherapy on the oral microbiome. We aimed to characterize changes in the oral bacterial microbiome based on 16S rRNA gene amplicon analysis during chemotherapeutic treatment, as a potential target to treat common oral side effects occurring during therapy. The chemotherapeutic drugs used in our study for patient treatment were trastuzumab, docetaxel, pertuzumab, epirubicin, and cyclophosphamide. We find a significant increase in the relative abundance of potentially pathogenic taxa like *Escherichia/Shigella* and non-significant trends in the relative abundance of, for example, *Actinomyces* ssp. In conclusion, the role of microbiota in the oral side effects of chemotherapeutic treatment needs to be considered and should be analyzed in more detail using larger patient cohorts. Oral side effects in breast cancer patients undergoing chemotherapy are a common burden and should be treated for a better tolerability of the therapy.

## Introduction

Deep molecular analyses in the age of personalized medicine is poised to improve the management of multiple diseases worldwide, enabling enhanced treatment responses of so-far deadly illnesses (1). Nevertheless, in addition to the desired effects of pharmaceutical mechanisms, most substances and in particular anticancer therapies elicit minor-to-severe adverse effects. Cancer therapies including chemotherapy, targeted therapies, and radiotherapy have evolved during the last decades, to become more effective and tolerable. However, patients still suffer from a multiplicity of oral, intestinal, neurologic, systemic, and many more severe adverse effects in response to cytotoxic and cytostatic drugs that interfere with the cell cycle and cell proliferation (2). Breast cancer, a disease that is still the leading cause of cancer death in women worldwide (3), is regularly treated with chemotherapeutics. Adjuvant and neoadjuvant therapy options depend on the distinct histological subtype, molecular tumor biology, and clinical stage (4). Generally, recommended treatment options are chemotherapeutical substances such as anthracycline, taxane, platinum, and capecitabine as well as trastuzumab, pertuzumab, emtansine, and neratinib for antihuman epidermal growth factor receptor 2 therapy (4). Standard endocrine therapy includes tamoxifen, aromatase inhibitor, and ovarian ablation or suppression (4). Classifications are based on the expression of the hormone receptor and human epidermal growth factor receptor 2. Additionally, subtypes are determined by the expression level of progesterone and estrogen receptors. Furthermore, tumor heterogeneity is found at the molecular level as genomic testing has revolutionized and highly individualized therapy decisions (5–7). Regardless of how much a chemotherapeutic treatment strategy is weighed and tailored to individual needs, treated patients suffer from a variety of severe oral phenotypes, among the aforementioned adverse effects. These oral complaints vary from altered taste sensation, xerostomia, osteonecrosis of the jaw, or mucositis that may affect the whole gastrointestinal tract (8, 9) . The probability of the development of such severe side effects depends on the dose and frequency of the used drug, the drug itself, a patient’s general health condition (e.g., malnutrition, preexisting diseases, and oral health) and age (10). Painful oral inflammation severely restricts the quality of life of patients and can cause therapy discontinuation with life-threatening consequences for the patients (11, 12). In addition to the cytotoxic effects of therapeutically drugs, often, superinfections with oral pathogens like *Actinomyces* ssp. are detected in the patients (2, 13). To overcome or at least manage these problems, clinicians and researchers developed guidelines for the treatment of chemotherapy-induced side effects for nausea and vomiting (14), while little progress is made to date in the treatment of the oral complications. For the use of tissue repair upon mucotoxic injury, platelet-rich plasma, as a source of growth factors, has been suggested (15). Similarly, glutamine was proposed as an important factor for mucosal healing during and after chemotherapy (16). Natural substances like propolis (15), curcumin (17), or honey (18) were tested as therapeutics to ameliorate the cytotoxic and cytostatic side effects. A recent review summarized studies on natural substances derived from *Plantago major*, *Isatis indigotica*, or *Olea europaea* with anti-inflammatory effects, which have been successfully tested in clinical trials to treat oral mucositis (19, 20).

Moreover, the effects of the oral microbiome on oral and dental health are well characterized (21, 22). Upon the dysbiosis of the oral community, pathogenic taxa like *Porhyromonas*, *Prevotella*, *Veillonella*, or *Actinomyces* my take over and cause critical illnesses (13, 23). On the contrary, beneficial microbes can act anti-inflammatory as already described for intestinal diseases (24). The successful application of orally administered probiotics in a mouse model of intestinal mucositis may reflect a promising approach to prevent or treat the oral adverse effects of chemotherapy (25). A prerequisite for the microbiome-related treatment options of severe oral phenotypes is the identification of chemotherapy-related changes in the microbial pattern of patients.

In this prospective study, we analyzed the saliva microbiome of 20 chemotherapeutically treated (and in addition with monoclonal antibodies and hormones) breast cancer patients before, during, and after the onset of therapy. We aimed to analyze (1) the oral microbial community and potential changes to the community pattern upon chemotherapy in breast cancer patients and (2) a potential role of *Actinomyces* ssp. during the course of therapy as a suspected pathogen for the development of oral diseases (26). We suggest that chemotherapy in general changes the oral microbiome as already demonstrated and reviewed for the gut microbiome (27). The characterization of the chemotherapy-induced changes of the oral microbiome might be an important tool to develop new therapeutic strategies to overcome stressful side effects for patients.

## Material and methods

### Sample collection

For this study, we collected oropharyngeal saliva from 20 patients older than 18 years, diagnosed with non-metastatic breast cancer, at three time points: (T1) the first day of chemotherapy, which was scheduled for 5–6 months, (T2) after 3 months from the start of chemotherapy, and (T3) 1 year (52 weeks) after the start of chemotherapy (for details, refer to **Table 1**). All patients suffered from breast cancer with a histological subtype of “ductal.” None of them had metastasis. They were all treatment naive early-stage breast cancer patients, who were scheduled for standard therapy (neoadjuvant chemotherapy). For chemotherapy, the substances epirubicin, cyclophosphamide, docetaxel, trastuzumab, and pertuzumab were used according to the current National Comprehensive Cancer Network guidelines (https://www.nccn.org/; last access April 2022) and therapeutic schemes are shown in detail in **Table 1**. Diagnosed patients were scheduled for neoadjuvant chemotherapy (mean age ± SD: 51.7 ± 11 years), and all gave written, informed consent to participate in the study. Exclusion criteria for participants were: (i) the history of a metastatic disease and/or having received prior chemotherapy, (ii) the use of antibiotics within 1 month prior to enrollment, (iii) the use of bisphosphonates, denosumab, or oral hormone contraceptives within 3 months prior to enrollment, (iv) having undergone to immunosuppressive treatments, and (v) a known endocrine disorder requiring treatment (**Table 1**). The study was performed in accordance to the Declaration of Helsinki and good practical guidelines and was approved by the local ethics committee (EK1539/2016) of the Medical University of Vienna. All samples were frozen in 2-ml tubes immediately after collection in liquid nitrogen and stored at -80°C until nucleic acid isolation.

**Table 1:** Detailed clinical information and patient annotations on the 20 breast cancer patients included in the study.

Patient ID	Age	HER2	Estrogen receptor	Progesterone receptor	Neoadjuvant chemotherapy*
*001GY*	49	3+	positive	positive	EC –> DTP
*002GY*	41	negative	negative	negative	Dtx –> EC
*003GY*	43	negative	positive	positive	EC –> Dtx
*005GY*	52	negative	negative	negative	EC –> Dtx
*007GY*	65	3+	positive	positive	EC –> DTP
*009GY*	42	3+	positive	positive	EC –> DTP
*010GY*	36	negative	positive	negative	EC –> Dtx
*011GY*	60	3+	positive	negative	EC –> DTP
*013GY*	63	negative	positive	negative	EC –> Dtx
*014GY*	64	negative	positive	positive	EC –> Dtx
*015GY*	46	3+	negative	negative	DTP –> EC
*016GY*	64	negative	positive	positive	EC –> Dtx
*017GY*	58	negative	positive	negative	EC –> Dtx
*018GY*	33	negative	positive	positive	EC –> Dtx
*019GY*	62	negative	positive	positive	EC –> Dtx
*022GY*	44	3+	negative	negative	EC –> DTP
*023GY*	60	3+	positive	positive	EC –> DTP
*025GY*	31	negative	positive	positive	Dtx –> EC
*026GY*	52	negative	negative	negative	Dtx –> EC
*027GY*	65	3+	negative	negative	EC –> DTP

*EC, epirubicin + cyclophosphamide; DTP, docetaxel + trastuzumab + pertuzumab; Dtx, docetaxel.

### Total DNA isolation, 16S rRNA gene amplification, and sequencing

For total DNA isolation, frozen samples were thawed, treated with DTT (50 µg/ml at 37°C for 20 min) and 250 µl of the sample was used for isolation according to published protocols in a MagnaPure LC device (Roche, Mannheim, Germany) with the MagnaPure LC DNA III Isolation Kit (Bacteria, Fungi) (Roche, Mannheim, Germany) (28). DNA was eluted in 50-µl elution buffer (Roche, Mannheim, Germany) and stored at -20°C till analysis. For 16S rRNA gene PCR, 2 µl of template DNA were used in a standard PCR setup in 25 µl reactions in triplicates with Roche High Fidelity Polymerase (Mannheim, Germany) and the target-specific primers 515F (GTGYCAGCMGCCGCGGTAA) and 926R (CCGYCAATTYMTTTRAGTTT), 30 amplification cycles, according to published protocols (28). The indexing of samples, pooling, purification, and quality control were performed as published elsewhere, and the final library was sequenced at 9pM with 20% PhiX on an Illumina MiSeq desktop sequencer (Illumina, Eindhoven, Netherlands) with v3 600 cycles chemistry in 2×300 sequencing mode. FASTQ files were used for data analysis, and raw data were archived in the European Nucleotide Archive (ENA) and can be downloaded with the accession number PRJEB51689 (https://www.ebi.ac.uk/ena/; last access March 2022).

### Data analysis

Paired end raw sequence reads were analyzed with the Quantitative Insights Into Microbial Ecology 2 (QIIME2 2018.4) tool according to standard settings described in the QIIME2 moving picture tutorial (29). Quality filtering, denoising, dereplicating, and chimera filtering with default settings were performed using the DADA2 plugin of QIIME2 (30). Taxonomic classification was achieved by using the QIIME q2-feature-classifier and a pre-classified SILVA database (version 138) at a 99% sequence identity level (31). Feature and taxonomy tables were used for further statistical analysis with MicrobiomeExplorer v1.4.0 (32) on R version 4.1.3. (33) with default settings. The minimal read number was set to 8,200 reads per sample; for blank values, roll down taxonomy was used and cumulative sum scaling (CSS) for normalization. Pairwise t-test, DeSeq2, and Kruskal–Wallis tests were used for differential abundance analysis to compare microbiome composition across time points. Correlation analysis for microbiota was performed with Spearman rank correlation analysis (34). For longitudinal analyses, we additionally used the mixed-effect model Microbiome Multivariable Association with Linear Models2 (MaAsLin2) in R as recently published (35, 36) to evaluate significant differences in alpha and beta diversity and differential abundance.

## Results

In this prospective study, we performed the collection and metagenomics sequencing of 60 oral samples (20 patients with chemotherapy at three timepoints) on an Illumina Miseq desktop sequencer. Sequencing revealed a total of 6,929,221 raw reads (min. reads 776, max. reads 172,814 per sample, +/- 30,228) and after all quality-filtering steps, denoising, and the removal of chimeras, a total of 1,601,511 reads were used for data analysis (min. reads 16, max. reads 109,706, +/- 4,297). One sample with only 16 reads (T1 group) was removed from further data analysis, and statistical analysis was performed according to the three time points of therapy. The quality metrics of reads were performed in MicrobiomeExplorer according to default settings (**Supplementary Figure S1**).

### Alpha diversity calculations

**Figure 1:**
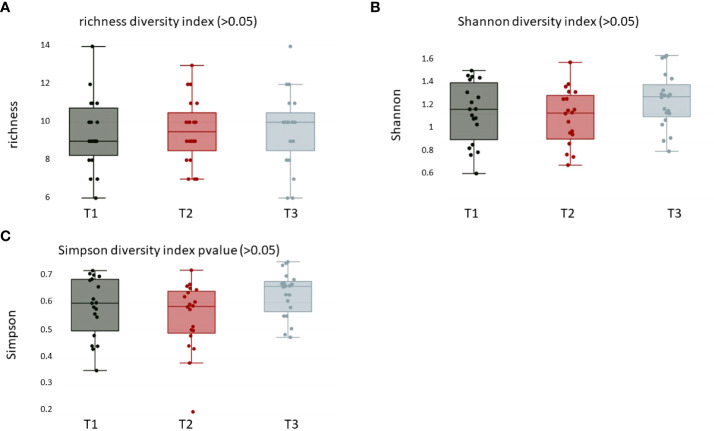
Alpha diversity calculations over all groups of **(A)** richness (pvalue > 0.05), **(B)** Shannon (pvalue > 0.05), and **(C)** Simpson (pvalue > 0.05) diversity index at the phylum level. T1: before/at start of chemotherapy, T2: 12 weeks after the onset of chemotherapy, T3: 52 weeks after the onset of chemotherapy.

**Figure 2:**
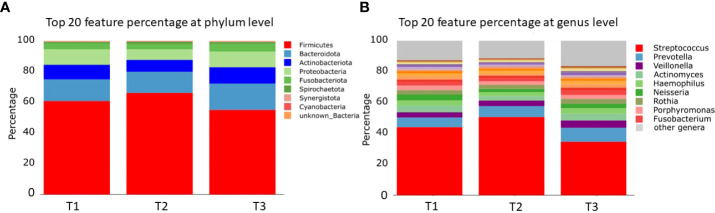
Bar charts of relative microbial abundance at the **(A)** phylum and **(B)** genus levels per sample group (the 20 most abundant taxa are shown). T1: before/at start of chemotherapy, T2: 12 weeks after the onset of chemotherapy, T3: 52 weeks after the onset of chemotherapy.

For alpha diversity calculations on the phylum and genus levels, we calculated, after CSS normalization, Shannon diversity index (pvalue >0.05), Simpson (pvalue >0.05), and richness (pvalue >0.05). None of the performed alpha diversity calculations revealed statistical significant differences between the three sample groups (**Figures 1A–C**). The phyla with highest relative abundance in all samples at all timepoints were Firmicutes (T1: 59%, T2: 69%, T3: 54%), followed by Bacteroidota (T1: 15%, T2: 15%, T3: 18%), Actinobacteriota (T1: 8%, T2: 8%, T3: 10%), Proteobacteria (T1: 10%, T2: 7%, T3: 10%), Fusobacteriota (T1: 4%, T2: 4%, T3: 5%), and Spirochaetota (T1: 1%, T2: 2%, T3: 1%). All other detected phyla (Synergistota, Cyanobacteria, Desulfobacterota, and unknown bacteria) were present in the samples with less than 1% (**Figure 2A**, **Supplementary Table S1**). The most abundant genera detected were *Streptococcus*, *Prevotella*, and *Veillonella* (**Figure 2B**, **Supplementary Table S1**).

### Beta diversity calculations

**Figure 3:**
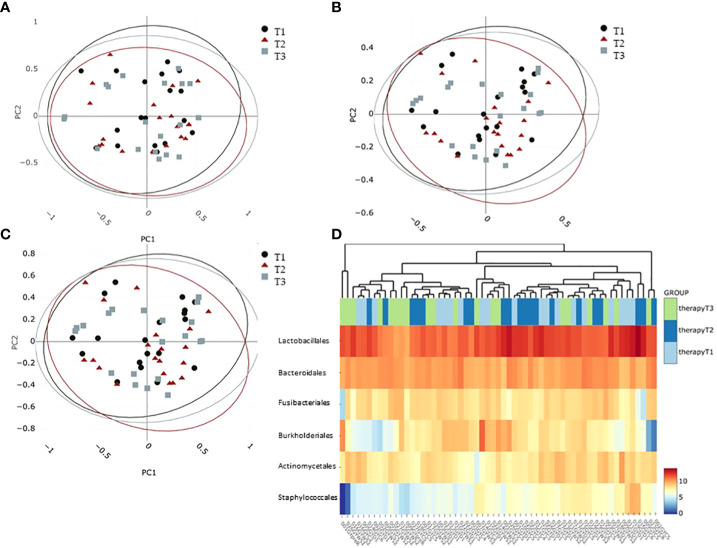
Beta diversity calculations at phylum level characterizing the intersample difference: **(A)** Chao, **(B)** Bray–Curtis as well as **(C)** Jaccard indices. Confidence ellipses indicate a 95% confidence level. **(D)** Abundance heatmap plotting to visualize hierarchical clustering on the six most dominant taxa at the order level. Colors from blue to red indicate feature abundance in the log scale. T1: before/at start of chemotherapy, T2: 12 weeks after the onset of chemotherapy, T3: 52 weeks after the onset of chemotherapy.

Oral microbiome beta diversity calculations over all samples of breast cancer patients were characterized by bacterial diversity indices (Adonis on Chao, Bray–Curtis, and Jaccard; pvalues = 0.756, 0.721, and 0.747, respectively) at the phylum level comparing the different time points (**Figures 3A–C**) and did not reveal statistically significant differences between the groups. Calculations on class, order, family, and genus levels did not reveal significant differences either. Heat map analysis on the logScale of normalized read counts at the order level clearly exhibited the dominance of Lactobacillales in all oral samples (**Figure 3D**) but did not reveal clustering according to sample groups (**Figure 3D**). Interestingly, the order *Burkholderiales* showed a trend to build a cluster of eight samples compared to all others as well as the order *Staphylococcales* was less abundant in approximately half of the samples (**Figure 3D**).

### Differential taxa abundance

**Figure 4:**
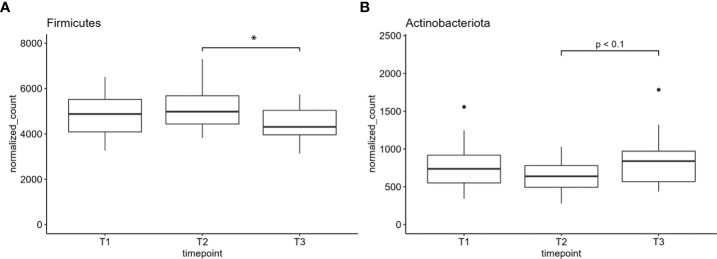
**(A)** The normalized counts of the phylum Firmicutes and **(B)** Actinobacteriota. Normalized counts are plotted; *indicates pvalue < 0.05. T1: before/at start of chemotherapy, T2: 12 weeks after the onset of chemotherapy, T3: 52 weeks after the onset of chemotherapy.

**Figure 5:**
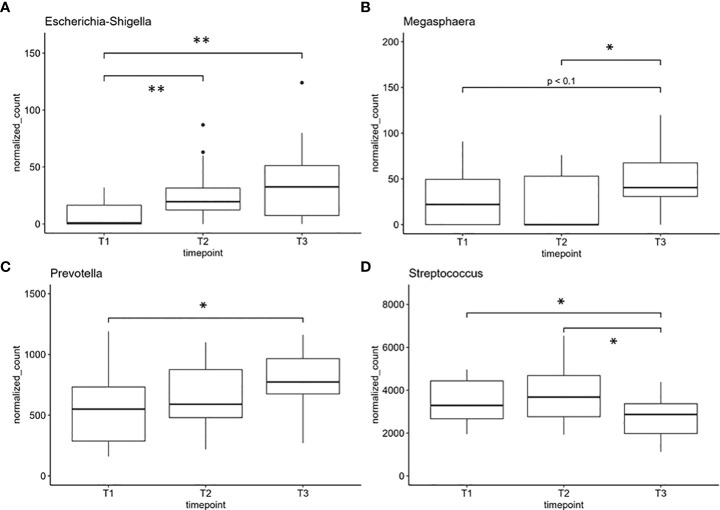
Genera with significant differences in normalized counts between sample groups: **(A)** Escherichia/Shigella, **(B)** Megasphaera, **(C)** Prevotella, and **(D)** Streptococcus, one of the most abundant genera. *indicates pvalue < 0.05. **indicates pvalue < 0.01. T1: before/at start of chemotherapy, T2: 12 weeks after the onset of chemotherapy, T3: 52 weeks after the onset of chemotherapy.

Finally, we performed differential analysis at phylum and genus levels over all samples between the three time points and found one phylum and four genera to be significantly altered between the sample groups (**Figures 4**, **5**). Further, one phylum and three genera showed at least a non-statistically significant trend of difference between the timepoints (**Figure 4** and **Supplementary Figure S2**). The phylum Firmicutes decreased significantly in its abundance between 12 and 52 weeks after onset of the chemotherapy. No difference was found for Firmicutes after 12 weeks after the onset of treatment (**Figure 4A**). *Actinobacteriota* showed a non-significant trend of reduction in the number of normalized counts after 12 weeks and an increase after 52 weeks and return to the basal values (**Figure 4B**). On the genus level, *Escherichia/Shigella*-normalized read counts differed highly significant between T1 and T2 (pvalue <0.01) as well as between T1 and T3 (pvalue <0.01) (**Figure 5A**). No significant differences were observed between timepoint T2 and T3. The genus *Megasphaera* increased between T2 and T3 (pvalue <0.05) with a non-significant elevation between T1 and T3 (pvalue <0.1) (**Figure 5B**). The read counts of *Prevotella* increased from T1 to T3 (pvalue <0.05), and *Streptococcus*, the genus dominating the microbial community in all sample groups, decreased significantly at T2 and T3 compared to T1 (**Figure 5D**). Non-significant trends were found in the genera *Actinomyces*, *Dialister*, and *Stenotrophomonas* (**Supplementary Figure S2**). *Actinomyces* slightly increased between T2 and T3, whereas the number of reads were elevated between T1 and T3 in the genus *Dialister*. *Stenotrophomonas* was nearly absent at T1 in most patients but successively increased over time until T3 (**Supplementary Figure S2**). All normalized feature count data are made available for download in **Supplementary Table S1**.

### Spearman correlation analysis

The Spearman correlation analysis of the significantly altered genus *Escherichia/Shigella* revealed a significant correlation with *Elizabethkingia* (pvalue=0.02, rho=0.516), *Actinomyces* (pvalue=0.008, rho=-0.578), and *Megasphaera* at T2 (pvalue=0.029, rho=-0.488) as well as *Porphyromonas* at T3 (pvalue=0.014, rho=-0.542) (**Supplementary Figure 3**).

### Longitudinal analysis

MaAsLin2 analyses did not reveal any significant associations of alpha (Shannon diversity, richness, und evenness) or beta (Bray–Curtis und Jaccard) diversity calculations and the time points T1, T2, and T3. Nevertheless, MaAsLin2 analysis revealed a significant positive association of Prevotellaceae with T1 and T3; pvalue <0.05) and a significant negative association of *Neisseria* with T1 and T3 (pvalue <0.05).

## Discussion

In this study, we characterized the salivary 16S rRNA gene–based microbial pattern of 20 breast cancer patients who underwent chemotherapy. The drugs used for therapy were Herceptin (trastuzumab), Taxotere (docetaxel), Perjeta (pertuzumab), epirubicin, and cyclophosphamide. All patients received the very same cytotoxic chemotherapy backbone with docetaxel, epirubicin, and cyclophosphamide. This combination has a distinct risk for oral mucositis [reviewed in Seiler etal. (37)] or (febrile) neutropenia and the mechanism of action. Since it would be unethical to administer those drugs as single agents in the neoadjuvant curative setting, the impact of those compounds on the oral mucositis or dysbiosis remains elusive. Additionally, the eight Her2-positive patients were exposed to the monoclonal antibodies trastuzumab and pertuzumab. The combination of trastuzumab or pertuzumab with chemotherapy does not significantly potentiate toxicity including stomatitis or neutropenia (38, 39) but enhances the efficacy by Her2 targeting. Interestingly, it was recently shown that the gut microbiome affects the efficacy of trastuzumab therapy (40). While it is unknown if this finding applies to the oral microbiome as well, there is no evidence that the opposite holds true, that is, trastuzumab therapy modulates the gut or oral microbiome. From this background, it seems evident that this was a homogenous patient population, which was exposed to the very same cytotoxic chemotherapy regimens. Although it cannot be ruled out that the sequence of the drugs and/or the addition of the monoclonal antibodies affected the outcome of our study, it is rather unlikely based on the data outlines above.

As chemotherapeutics affect not only the cancer cells but also healthy, rapidly proliferating cells (e.g., in hematopoiesis or mucous tissue), the oral cavity is frequently affected by severe injurious reactions. In addition, medication-related oral phenotypes are associated with infections mediated by microbial pathogens due to treatment-induced dysbiosis (2, 23). In mice, studies on probiotic strains such as *Streptococcus salivarius* K12 for the treatment of oral mucositis due to radiotherapy showed promising results. Oral ulcers were reduced, anaerobic pathogens were less abundant, and the epithelium and mucosa were of a healthier condition (41). Therefore, we aimed to identify changes in the abundance of classic oral pathogens due to chemotherapy as well as alterations in overall microbial patterns due to treatment- induced dysbiosis in a long-term scale before (baseline) (T1), during (T2), and 1 year after the onset of the treatment (T3). Overall, alpha and beta diversity metrics did not significantly differ between the three time points in our sample cohort. Nevertheless, we found a significant decrease in the abundance of the phylum Firmicutes between T2 and T3, indicating a long-term effect of chemotherapeutical treatment with a microbial dysbiosis even 1 year after chemotherapy. The absence of these changes compared to T1 might be due to our small sample cohort and/or the high level of interindividual variation in the abundance of Firmicutes. As many beneficial oral species belonged to the genus Firmicutes (e.g., *Streptococcus* sp. and *Lactobacillus* sp.), the decrease in this phylum might be an indication for their displacement and a hint for changes toward a more pathogenic oral microbiome. The abundance of all other phyla corresponded to previous publications on the oral microbiome (42). A trend to a significantly increased abundance of the phylum Actinobacteriota in T3 compared to T2 needs to be verified in a larger sample cohort.

An in-depth analyses of our results revealed a highly significant increase in the normalized counts of Escherichia/Shigella, Megasphaera, and Prevotella. Escherichia/Shigella is a common oral and intestinal pathogen (22) also associated with changes in the human microbiome after SARS-CoV-2 infections (43). Oral Megasphaera was associated with inflammatory illnesses like rheumatoid arthritis and periodontitis (44, 45). Their increase might be an indicator for the chronic inflammatory reaction by the damage to the epithelium. Prevotella is a common anaerobic oral bacterium that is also enriched in periodontitis, associated with early childhood caries, and described as potentially harmful for the mucosal surface (46). On the contrary, *Streptococcus*, the most abundant genus in the samples, decreased significantly during the treatment corresponding to the observed decrease of Firmicutes. In addition to pathogenic species, many *Streptococcus* species from the genus are known to be highly beneficial in the oropharyngeal community, preventing the host from oral infections, tonsillitis, and otitis media (47). Even a protective effect against viral infections is already described (48). The negative association of the genus *Neisseria* over time was revealed by MaAsLin2 analysis. The representatives of the genus *Neisseria* are common, mostly aerobic taxa in the human oropharynx. Most of them are not pathogenic with some prominent pathogenic exceptions (*N. gonorrhoeae*, *N. meningitides*, or *N. sicca*). We hypothesize that the genus belongs to the normal oral microbiome and becomes reduced upon chemotherapeutic treatment as a hint of oral microbial dysbiosis as the oral community is suspected to be a main driver for oral health and disease (22). Further, other than the taxa described, we do not find any evidence of an increased abundance of *Actinomyces* ssp. upon chemotherapeutic treatment. Larger studies on the oral and intestinal changes upon chemotherapy, radiation, and several types of medication and the different associated phenotypes (mucositis, osteonecrosis of the jaw) will be required to tailor the adequate treatment. Probably, nutritional supplementations of pre- and probiotics might be a therapeutic tool to rebalance chemotherapeutically induced microbial dysbiosis or pathogenic oral phenotypes for a better tolerance of vital therapies.

In conclusion, our saliva data on a cohort of breast cancer patients suggest the development of dysbiosis in the oral microbiome upon chemotherapeutic treatment. Recent state-of-the-art studies suggest a significant involvement of the microbiome not only in the development and treatment of cancer (49) but also in the adverse effects of cancer therapy (41). Therefore, the determination of the status quo from patients’ oral and intestinal microbiomes should be considered routinely during chemotherapy. In a future horizon, the administration of next-generation probiotics, prebiotics, or postbiotics as nutritional supplementation should be considered for all patients with oral complications as the side effects of chemotherapy treatment, to support a healthy oral microbiome and decrease the side effects of medication.

## Data availability statement

The datasets presented in this study can be found in online repositories. The names of the repository/repositories and accession number(s) can be found below: https://www.ebi.ac.uk/ena, PRJEB51689.

## Ethics statement

The study was performed in accordance to the Declaration of Helsinki and good practical guidelines, and was approved by the local ethics committee (EK1539/2016) of the Medical University of Vienna. The patients/participants provided their written informed consent to participate in this study.

## Author contributions

IK, CB, IB, HS, CSi, TF, and CSt designed and performed the experiments, analyzed the data, and wrote the manuscript. AM, CH analyzed data, contributed materials, and reviewed the manuscript. AP provided expertise and feedback, and wrote and reviewed the manuscript. All authors contributed to the article and approved the submitted version.

## Funding

This research was funded by the Institute of Microbiome Research at the Karl-Landsteiner Society and the Austrian Science Funds, P28102-B30 and P 32169-B30.

## Conflict of interest

The authors declare that the research was conducted in the absence of any commercial or financial relationships that could be construed as a potential conflict of interest.

## Publisher’s note

All claims expressed in this article are solely those of the authors and do not necessarily represent those of their affiliated organizations, or those of the publisher, the editors and the reviewers. Any product that may be evaluated in this article, or claim that may be made by its manufacturer, is not guaranteed or endorsed by the publisher.

## Supplementary material

Supplementary Table 1 Feature Counts from phylum to species level.
