# Supplementary figures and images for: Chemotherapy-associated oral microbiome changes in breast cancer patients
Source: Front Oncol. 2022 Aug 9;12:949071. doi: 10.3389/fonc.2022.949071 (PMC9396302; doi:10.3389/fonc.2022.949071)

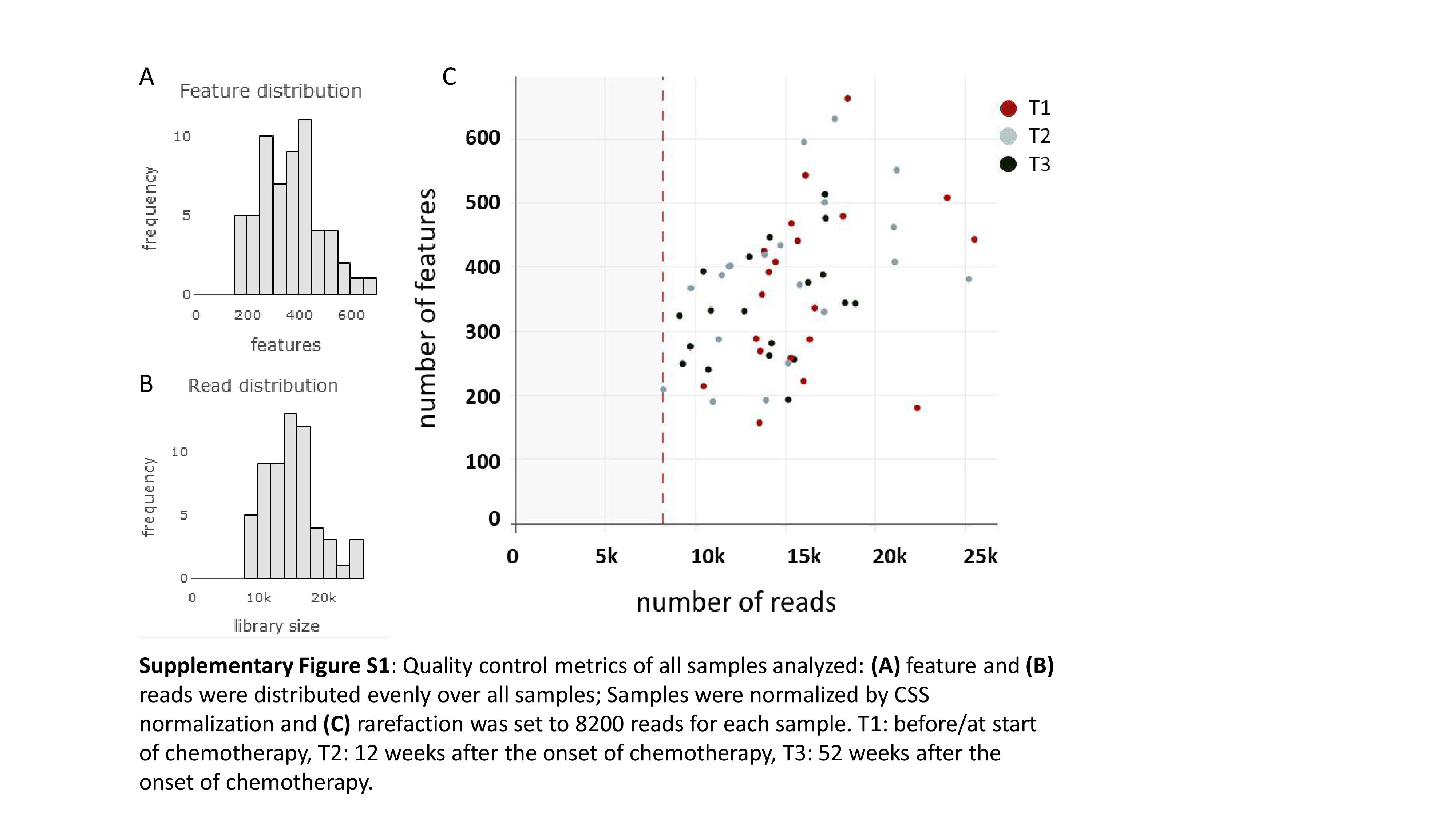

Supplement: Supplementary Table 1 — Feature Counts from phylum to species level. [file Image_1.jpeg]

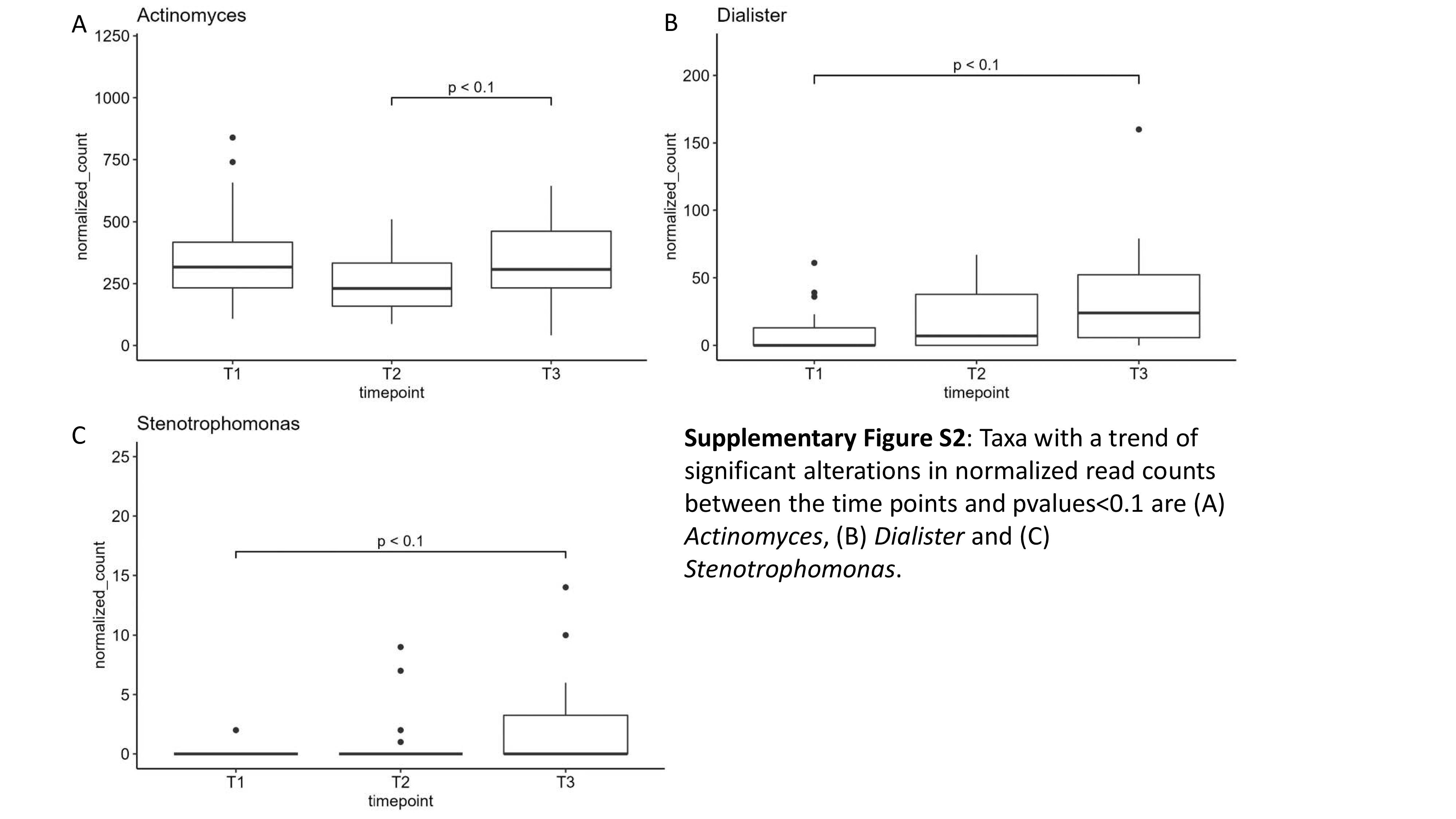

Supplement: Supplementary file 2 [file Image_2.jpeg]

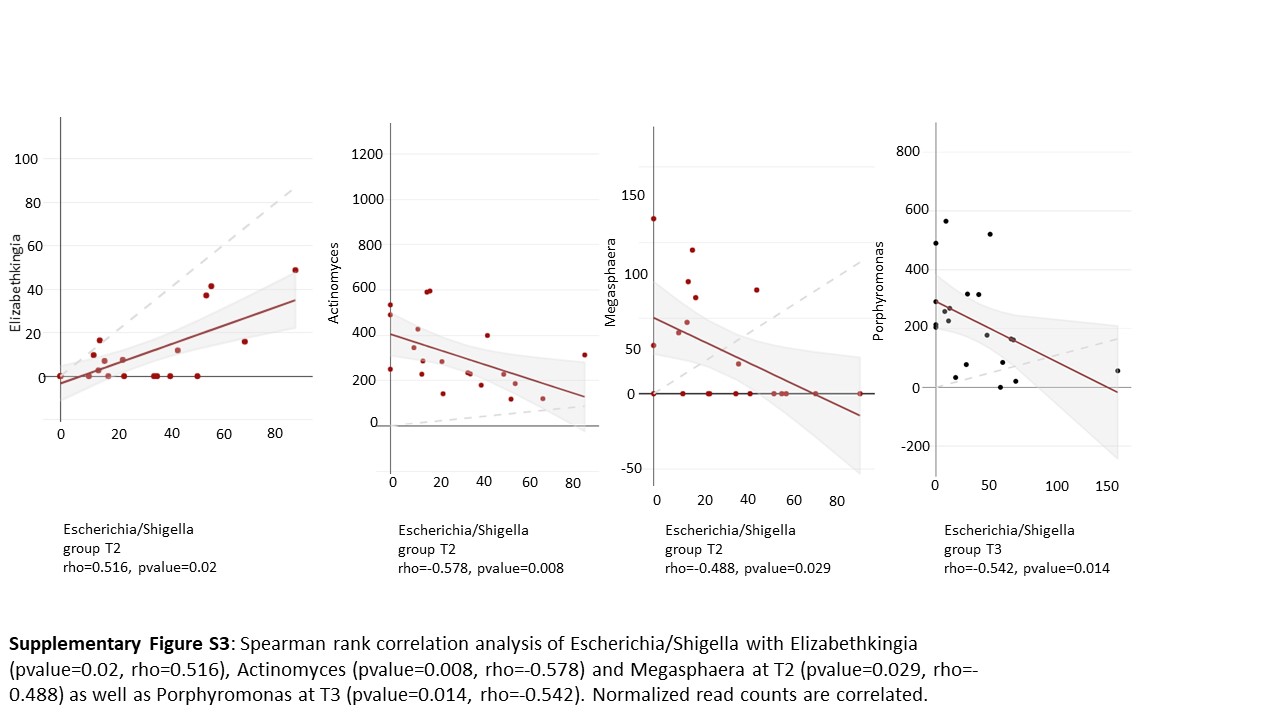

Supplement: Supplementary file 3 [file Image_3.jpeg]
